# CFD Simulations of an Air-Water Bubble Column: Effect of Luo Coalescence Parameter and Breakup Kernels

**DOI:** 10.3389/fchem.2017.00068

**Published:** 2017-09-21

**Authors:** Alizeb Hussain Syed, Micael Boulet, Tommaso Melchiori, Jean-Michel Lavoie

**Affiliations:** ^1^Industrial Research Chair on Cellulosic Ethanol and Biocommodities, University of Sherbrooke Sherbrooke, QC, Canada; ^2^Enerkem Sherbrooke, QC, Canada

**Keywords:** population balance model (PBM), bubble size distribution, time-average radial profiles of holdup and axial liquid velocity, bubble column

## Abstract

In this work, CFD simulations of an air-water bubbling column were performed and validated with experimental data. The superficial gas velocities used for the experiments were 0.019 and 0.038 m/s and were considered as an homogeneous regime. The former involves simpler physics when compared to a heterogeneous regime where the superficial velocities are higher. In order to simulate the system, a population balance model (PBM) was solved numerically using a discrete method and a closure kernels involving the Luo coalescence model as well as two different breakup models: Luo's and Lehr's. For the multi-phase calculations, an eulerian framework was selected and the interphase momentum transfer included drag, lift, wall lubrication, and turbulent dispersion terms. A sensitivity analysis was performed on a Luo coalescence kernel by changing the coalescence parameter (*c*_0_) from 1.1 to 0.1 and results showed that the radial profiles of gas holdup and axial liquid velocity were significantly affected by such parameter. From the simulation results, the main conclusions were: (a) A combination of the Luo coalescence and Luo breakup kernels (Luo-Luo) combined with a decreasing value of *c*_0_ improves the gas holdup profiles as compared to empirical values. However, at the lowest value of *c*_0_ investigated in this work, the axial liquid velocity deteriorates with regards to experimental data when using a superficial gas velocity of 0.019 m/s. (b) A combination of the Luo coalescence and Lehr breakup models (Luo-Lehr) was shown to improve the gas holdup values with experimental data when compared to the Luo-Luo kernels. However, as *c*_0_ decreases, the Luo-Lehr models underestimate the axial liquid velocity profiles with regards to empirical values. (c) A first and second order numerical schemes allowed predicting similar radial profiles of gas holdup and axial liquid velocity. (d) The mesh sensitivity results show that a 3 mm mesh size can be considered as reasonable for simulating experimental data. (e) The inclusion of wall lubrication parameter was found to be significant, although only when using finer meshing. In addition, it allows an improvement of the axial liquid velocity at the core of the bubble column.

## Introduction

Bubble columns have reportedly been used in the chemical, petrochemical, bioprocesses, and pharmaceutical industries. In simple bubble column reactors, the gas phase is dispersed into a liquid or liquid-solid continuous phase. In general, depending on superficial velocities and column diameter, the regime inside the bubble column is either homogeneous, transitional, or heterogeneous (Deckwer, 1992). The former involves simpler physics as compared to the latter and most of the models (interphase, coalescence and breakup) were developed in that regime before being later implemented in the heterogeneous regime. The gas holdup as been reported as the most important design criterion in bubble columns. The latter is related to the bubble size, which ultimately allows determining the interfacial area and ultimately, defines the mass transfer phenomena. In biphasic non-reactive bubbly flows, the bubble size varies due to the gas and liquid velocities, inlet geometry, bubble coalescence, bubble breakup, and bubble growth, hence complicating the hydrodynamic behavior inside the system (Fan, 1989; Yeoh et al., 2014). Furthermore, the gas and liquid flow need closure terms in the interphase momentum transfer equations that depend locally on the velocity profiles, physical properties of phases and on the turbulence parameters that are still under development in the open literature (Ishii and Hibiki, 2011). Hence, a comprehensive understanding of the fluid dynamics is required and the latter would in turn be very useful in many industrial fields. Many researchers have used computational fluid dynamics (CFD) techniques to simulate biphasic bubble columns. The latter are in most cases simulated by the Euler-Euler approach (two-fluid) due to a lesser computational cost when compared to the Euler-Lagrange or volume of fluid (VOF) approaches. In gas-liquid flow, the interface involves both drag and non-drag forces.

The drag force has an influence on the macroscopic structure of the flow. For instance, the radial profiles of velocity and holdup depend on the drag coefficient, Reynolds number, Eotvos numbers, terminal velocity and on the physical properties of the continuous phase (Wang and Yao, 2016). Rzehak et al. (2017) simulated a bubbly flow in different operating conditions and the geometries using the Ishii drag coefficient (Ishii and Zuber, 1979) and the predicted results were reported to be in good agreement with experimental values. This drag correlation is suitable for a wider range of bubbles sizes and covers all flow regimes (homogeneous, transitional or heterogeneous).

In bubble columns, the shape of the radial profiles may change according to the net lateral lift force. According to Tomiyama (2004), small bubbles (*d*_*b*_ < 5.8 mm) have a positive lift coefficient and tend to go toward the reactor wall. However, larger bubbles have a negative lift coefficient and tend to stay at the core of the bubble column. Zhang et al. (2005, 2006) suggested that the inclusion of the Tomiyama lift coefficient could predict a better correlation with experimental values. Nevertheless, Masood and Delgado (2014) and Yamoah et al. (2015) studied the influence of wall lubrication force and found that the Tomiyama correlation (Tomiyama et al., 1995) tends to over-estimate the velocity profiles when compared to the Antal correlation (Antal et al., 1991) that however agrees well with experimental data. Finally, Lucas et al. (2007) developed a 1D-model, studying the effect of wall lubrication and turbulent dispersion forces and suggested that the combination of these non-drag forces provides reasonable results.

Laborde-Boutet et al. (2009) focused on different formulations of the turbulent model and showed that the Renormalization Group (RNG k-ε) predicts higher turbulent dissipation rate as compared to Standard k-ε, which is known to be underestimated (Jakobsen et al., 2005). Most of the coalescence and breakup kernels depend on the local turbulent dissipation rate. Therefore, Laborde-Boutet suggested that the RNG k-ε turbulent model should be used to implement a population balance model (PBM).

The bubble coalescence and breakup phenomenon requires a Population Balance Equations (PBE) which allows discretization into N-classes of bubble size that can then be coupled with a two-fluid model. A single momentum equation can be solved for all N-classes, an approach called Homogeneous multi size group (MUSIG). In the case of non-homogeneous multi size group (called iMUSIG), multiple momentum equations are solved, making the solutions computationally costly. Krepper et al. (2008) developed and worked on the simulation of a gas-liquid phase using an iMUSIG model and suggested that 2–3 subgroups are sufficient to capture the fluid behavior. It was concluded that although iMUSIG allows simulating the local radial profiles, it is still limited by breakup and coalescence kernels that use an isotropic turbulent approach. Similarly, Xu et al. (2013) simulated a bubble column using both MUSIG, and iMUSIG. The results showed that the former (which includes lift force) and the latter both agreed well with experimental results. Wang et al. (2003, 2005) studied the effect of different coalescence and breakup kernels and results showed that the Luo breakup (Luo and Svendsen, 1996) predicts lower breakup rates, while the Lehr kernel (Lehr et al., 2002) predicts higher breakup rates with regards to empirical values. The key difference in both kernels is in the estimation of the breakup efficiency. Luo's model includes the surface energy constraint, which shows that the break-up could only occur if the kinetic energy of the colliding eddies is higher than the surface energy necessary for bubble breakage. However, Lehr's model only considers the capillary constraint, assuming that the interfacial and inertial forces balance each other. Chen et al. (2005) also studied the effect of different kernels and concluded that the radial profiles were not sensitive as long as the breakup is increased 10 times. Xu et al. (2013) used Luo's model for bubble coalescence and breakup and modified the coalescence parameter to 0.5, generating results that were in good agreement with experimental data.

In literature, the most commonly used kernel for bubble coalescence is the Luo's model while for bubble breakup, the Luo and Lehr model are usually preferred. Luo's coalescence model over-predicts the collision frequency and needs adjustment to reduce the coalescence rate which can be achieved by tuning the coalescence parameter (Wang et al., 2005; Yeoh et al., 2014). Finally, the effect of coalescence parameter in Luo's model was not extensively reported in the open literature and only a handful of studies have been published so far (such as Xu et al., 2013, 2014). Furthermore, the influence of this coalescence parameter on radial profiles of gas holdup and axial liquid velocity using two different bubble breakup models is limited.

In light of this, this work intends to fill the gaps identified in the previously reported approaches with the specific target to fit with industrial applications. Hence, the main objectives of this paper are as follows:

▪ Investigate the influence of the coalescence parameter on radial profiles using a combination of Luo coalescence and Luo breakup models.
▪ Study the influence of the coalescence parameter on radial profiles using a combination of Luo coalescence and Lehr breakup models.
▪ Perform a sensitivity analysis of a number of bubble classes and numerical schemes.
▪ Provide a sensitivity analysis of the wall lubrication force and the mesh sizes.

The presented results for a biphasic bubbling column were generated on a 2D-axisymmetric geometry and the predicted time-averaged profiles were compared with the literature data of Hills (1974). CFD-simulations were conducted using the commercial software ANSYS-Fluent v.17.2.

## Model development

### Two-fluid model and interphase

The eulerian framework was considered for the air-water system for which the conservation equations were solved for each phase while the mass and momentum equations are reported below as:

(1) $$\frac{\partial (\alpha_{q}\rho_{q})}{\partial t}+\;\nabla .\left(\alpha_{q}\rho_{q}u_{q}\right)=0$$

(2) $$\begin{array}{l} \frac{\partial (u_{q}\rho_{q}\alpha_{q})}{\partial t}+\;\nabla .\left(\alpha_{q}\rho_{q}u_{q}u_{q}-\mu_{q}\alpha_{q}(\nabla u_{q}+{\left(\nabla u_{q}\right)}^{T})\right)\\ \;=\;-\alpha_{q}.\nabla P+\;F_{pq}+\alpha_{q}\rho_{q}g \end{array}$$

#### Drag force

The drag force is in this case generated by the slip velocity between the gas and liquid phases, which depends on the drag coefficient as well as the interfacial area of bubbles. For this study, the drag coefficient involves the Ishii correlation (Ishii and Zuber, 1979), which considers a wide range of bubble size, varying according to the flow regime (viscous, distorted and capped regime). This variation of the flow regime depends in turn on the local Reynolds number in the viscous regime (0 ≤ *Re* < 1, 000) and for distorted and cap regime (*Re* ≥ 1, 000). The drag force and the Ishii drag coefficient are given by:

(3) $$F_{D}=-\frac{3}{4}\alpha_{p}\rho_{q}\frac{C_{D}}{d_{p}}\left|u_{p}-u_{q}\right|\left(u_{p}-u_{q}\right)$$

For viscous regime [*C*_*D,dis*_ < *C*_*D,vis*_],

(4) $$C_{D,vis}=\frac{24}{Re}\left(1+0.1Re^{0.75}\right)$$

For distorted regime [*C*_*D,vis*_ < *C*_*D,dis*_ < *C*_*D,cap*_],

(5) $$C_{D,dis}=\frac{2}{3}d_{p}\sqrt{\frac{g\Delta \rho_{pq}}{\sigma }}\left(\frac{1+17.67f^{*\frac{6}{7}}}{18.67f^{*}}\right),\;f^{*}={\left(1-\alpha_{p}\right)}^{1.5}$$

For capped regime [*C*_*D,dis*_ > *C*_*D,cap*_],

(6) $$C_{D,cap}=\frac{8}{3}{\left(1-\alpha_{p}\right)}^{2}$$

The relative Reynolds number Re is defined as:

(7) $$Re=\frac{\rho_{q}\left|u_{p}-u_{q}\right|d_{p}}{\mu_{q}}$$

#### Lift force

In bubble columns, each upward moving bubble experiences a force perpendicular to the direction of its motion. This force is called *transverse* or *lift force* and is calculated by taking into account the disperse phase fraction, the density of the continuous phase, the relative velocity between phases, the velocity gradients as well as the lift coefficient. The lift coefficient plays an integral role on the radial profiles of gas holdup and on the liquid velocity. Small bubbles (*d*_*b*_ < 5.8 mm) are known to have a positive lift coefficient and bubbles tend to go toward the lowest gradient of liquid velocity (i.e., toward the reactor's wall). Larger bubbles (*d*_*b*_ > 5.8 mm) however, are associated to a negative value and tend to stay at the core of the bubble column (Tomiyama, 2004). The lift force and the Tomiyama lift coefficient are given as follows:

(8) $$F_{L}=-C_{L}\alpha_{p}\rho_{q}\left(u_{p}-u_{q}\right)\left(\nabla u_{q}\right)$$

(9) $$C_{L}=\{\begin{array}{ll} \min\left[0.288\;tanh\left(0.121Re,f\left(Eo^{\prime }\right)\right)\right],\; & for\;Eo^{\prime }\leq 4\\ f\left(Eo^{\prime }\right), & for\;4\;\leq Eo^{\prime }\leq 10\\ -0.27 & for\;10\leq Eo^{\prime } \end{array}$$

Where,

(10) $$f\left(Eo^{\prime }\right)={0.00105Eo^{\prime }}^{3}\text{​}-\text{​}{0.0159Eo^{\prime }}^{2}-0.0204Eo^{\prime }+0.474$$

The modified Eotvos number *Eo*′ is defined as:

(11) $$f\left(Eo^{\prime }\right)=\frac{g\left(\rho_{q}-\rho_{p}\right)d_{h}^{2}}{\sigma }$$

Where,

(12) $$d_{h}=\frac{d_{b}\left(1+0.163Eo^{0.757}\right)}{\sigma }$$

The Eotvos number Eo is described as:

(13) $$Eo=\frac{g\left(\rho_{q}-\rho_{p}\right)d_{b}^{2}}{\sigma }$$

#### Wall lubrication force

The wall lubrication force acts near the vicinity of the wall and tends to push the bubbles away from it (Yeoh et al., 2014). The wall lubrication coefficient (Antal et al., 1991) depends mainly on the wall distance and the bubble size and it is given as:

(14) $$F_{WL}=C_{WL}\alpha_{p}\rho_{q}{\left|{\left(u_{p}-u_{q}\right)}_{\|}\right|}^{2}\left(n_{w}\right)$$

(15) $$C_{WL}=max\left(0,\frac{C_{w1}}{d_{p}}+\frac{C_{w2}}{y_{w}}\right)$$

#### Turbulent dispersion force

The turbulent dispersion force accounts for the interaction between turbulent eddies and the disperse phase (i.e., bubbles). The latter disperses the bubbles from the most to the least concentrated regions. This force depends on the drift velocity and the gradient of the disperse phase (Simonin and Viollet, 1990) and it is given by:

(16) $$F_{TD,q}=-F_{TD,p}=C_{TD}K_{pq}\frac{D_{t,pq}}{\sigma_{pq}}\left(\frac{\nabla \alpha_{p}}{\alpha_{p}}-\frac{\nabla \alpha_{q}}{\nabla \alpha_{q}}\right)$$

### Turbulent model

The mixture Renormalization Group (RNG) k-epsilon model is written as:

(17) $$\begin{array}{l} \frac{\partial (\rho_{m}k)}{\partial t}+\;\nabla .\left(\rho_{m}u_{m}k\right)=\nabla .\left(\left(\mu_{m}+\frac{\mu_{t,m}}{\sigma_{k}}\right)\right)\nabla k\\ \;+\;G_{k,m}-\rho_{m}\varepsilon \end{array}$$

(18) $$\begin{array}{l} \frac{\partial (\rho_{m}\varepsilon )}{\partial t}+\;\nabla .\left(\rho_{m}u_{m}\varepsilon \right)=\nabla .\left((\mu_{m}+\frac{\mu_{t,m}}{\sigma_{\varepsilon }})\nabla \varepsilon \right)\\ \;+\;\frac{\varepsilon }{k}\left(C_{1\varepsilon }G_{\varepsilon ,m}-C_{2\varepsilon }\rho_{m}\varepsilon \right) \end{array}$$

### Population balance model (PBM)

The PBM was solved numerically using the class method for which the volume based bubble number density function is given as:

(19) $$\begin{array}{l} \frac{\partial }{\partial t}n_{i}+\nabla .\left(u_{i}n_{i}\right)=\sum_{d_{j}=d_{i}}^{d_{max}}\Omega_{B}\left(d_{j}:d_{i}\right)-\Omega_{B}\left(d_{i}\right)\\ +\sum_{d_{j}=d_{min}}^{\frac{d_{i}}{2}}\Omega_{C}\left(d_{j}:d_{i}-d_{j}\right)-\sum_{d_{j}=d_{min}}^{d_{max}-d_{i}}\Omega_{C}\left(d_{j}:d_{i}\right) \end{array}$$

The local gas volume fraction (or holdup) is defined as follows:

(20) $$\alpha_{g}=\sum_{i=1}^{N}n_{i}\frac{\pi }{6}d_{i}^{3}$$

The Luo coalescence kernel (Luo, 1993) is the product of the collision frequency and coalescence efficiency. The binary coalescence between two classes of bubbles (*d*_*i*_ and *d*_*j*_) is given as follows:

(21) $$\Omega_{C}\left(d_{i}:d_{j}\right)=c_{0}{\left(d_{i}+d_{j}\right)}^{2}{\left({d_{i}}^{2/3}+{d_{j}}^{2/3}\right)}^{1/2}\varepsilon^{1/3}n_{i}n_{j}\;exp\left\{-\frac{t_{c}}{t_{I}}\right\}$$

Here *c*_0_ is the adjustable coalescence parameter, which equals 1.1 in the Luo coalescence model. Other coalescence models (Lee et al., 1987; Prince and Blanch, 1990) used the same approach but varied the coalescence parameter from 1.1 to 0.28. According to many authors who published on this aspect (Xu et al., 2013, 2014), the Luo coalescence model over-predicts the collision frequency and requires adjustments. As mentioned earlier, the most commonly used breakup models are the Luo kernel (Luo and Svendsen, 1996) and Lehr kernel (Lehr et al., 2002). Both models predict breakup rate and daughter size distribution directly from the models, hence the distribution does not need to be provided as an input parameter. The total breakup rate is given as:

(22) $$\Omega_{B}\left(d_{i}\right)=\;\int_{0}^{0.5}\Omega_{B}\left(d_{i}:d_{j}\right)df$$

The binary bubble breakup according to Luo and Svendsen (1996) and Lehr et al. (2002) is defined, respectively as:

(23) $$\begin{array}{l} \Omega_{B}\left(d_{i}:d_{j}\right)=0.9238\varepsilon^{1/3}d_{i}^{-2/3}\alpha \;\\ \int_{\xi_{min}}^{1}\frac{{\left(1+\xi \right)}^{2}}{\xi^{\frac{11}{3}}}exp\left(\frac{12\sigma c_{f}}{\rho_{q}\varepsilon^{2/3}d_{i}^{5/3}}\xi^{-11/3}\right)d\xi \end{array}$$

(24) $$\begin{array}{l} \Omega_{B}\left(d_{i}:d_{j}\right)=\frac{1.19\sigma }{\rho_{q}\varepsilon^{1/3}d_{i}^{7/3}f^{1/3}}\\ \int_{\xi_{min}}^{1}\frac{{\left(1+\xi \right)}^{2}}{\xi^{\frac{13}{3}}}exp\left(\frac{2\sigma \;We_{crit}}{\rho_{q}\varepsilon^{2/3}d_{i}^{5/3}f^{1/3}}\xi^{-2/3}\right)d\xi \end{array}$$

## Numerical setup

All simulations were run on a 2D axis-symmetric geometry. The assumption for the 2D axis-symmetric could be reasonable since experimental data reported by Hills (1974) and Degaleesan (1997) showed that the time-averaged flow field produces a stationary axis-symmetric flow pattern, hence supporting the validity of the 2D model. Simulations were validated using the experimental data published by Hills (1974), which was shown to be robust and is often used by other authors (Krishna et al., 1999; Van Baten, 2000; Ekambara and Joshi, 2005). Hills data has been extensively cited in literature explaining why the model developed in this work was validated using these empirical values. The two-fluids involved in the experiments consisted of air (acting as disperse phase) and water (considered as the continuous phase). The superficial gas velocity was varied between 0.019 and 0.038 m/s, range in which an homogeneous regime could be achieved (Krishna et al., 1999). The diameter and height of the cylindrical column were of 0.138 and 1.38 m, respectively. The static liquid height was 0.9 m and all the experimental observations were performed at a 0.6 m height. The inlet geometry of the experimental setup consisted of a perforated plate with 61 holes which all had a 0.0004 m diameter. Due to the limitation associated with the mesh size and computational cost, the gas was assumed to be introduced uniformly from the bottom of the column. This assumption was supported by Buwa and Ranade (2002) where the influence of the sparger design using a perforated plate (actual experimental inlet with holes) and the sintered plate was investigated and was shown to induce no significant difference with regards to empirical data. They also concluded that a hole diameter of 0.8 mm requires in turn a very fine meshing in the simulations, making it computationally very expensive. Similarly, Chen et al. (2005) also simulated a bubble column using a sintered plate instead of a perforated plate and reported that it is not essential to use the actual experimental inlet configuration.

The boundary condition involves a uniform inlet bubble size which was calculated from Kumar's correlation and was obtained for diameters of 3.6 mm and 4.5 mm at superficial gas velocities of 0.019 and 0.038 m/s, respectively (Kumar et al., 1976). The outlet and wall include atmospheric pressure and non-slip boundary conditions, respectively. Gas was the only mixture introduced from the inlet (α_*p*_ = 1), while the 0.9 m static column height involved α_*q*_ = 1 and α_*p*_ = 0. Above this level (free board), the gas and water phase fractions were α_*q*_ = 0 and α_*p*_ = 1, respectively. The bubble volume of each class was calculated from the following formula ($v_{i+1}/v_{i}=2^{r}$), where r is the ratio factor which equals to 1, 2 … n. For all the simulations (except for the mesh sensitivity analysis), a third order upwind scheme was used to discretize the continuity equation while the rest of the transport equations were solved by a second order scheme (see Table 1). The mesh sensitivity analysis was performed using a first order scheme due to convergence issues that were faced when solving the transport equations with higher order schemes for finer mesh. Convergence problems were encountered when an adaptive time step approach was used. In such cases, solutions tended to diverge due to the variation of the time step, especially at the initial flow time. The fixed time step was well consistent in term of convergence. Hence, 1E-04 s time steps were used and guaranteed that the courant number for air and water velocities was <1. Once a statistically steady state was reached, a time-averaged sampling was calculated for 30 s.

**Table 1:** Boundary conditions, physical properties and numerical schemes used in the simulation work.

	**Boundary and physical conditions**	**Units**
Inlet	Velocity inlet	
Outlet	Pressure outlet	
Wall	Non-slip condition	
Pressure-velocity coupling	Coupled	
Bubble inlet size	3.6 and 4.5	mm
Time step	1.00E-04	s
Column diameter	0.138	m
Column height	1.38	m
Ug	0.019 and 0.038	m/s
Static loading height	0.9	m
	**Numerical schemes for all the simulations**	**Numerical schemes only for the mesh sensitivity**
Continuity	QUICK	First order upwind
Momentum	Second order upwind	First order upwind
Turbulent model	Second order upwind	First order upwind
PBM	Second order upwind	First order upwind
Transient formulation	Second order implicit	First order upwind

## Results and discussion

### Number of classes comparison

**Figure 1:**
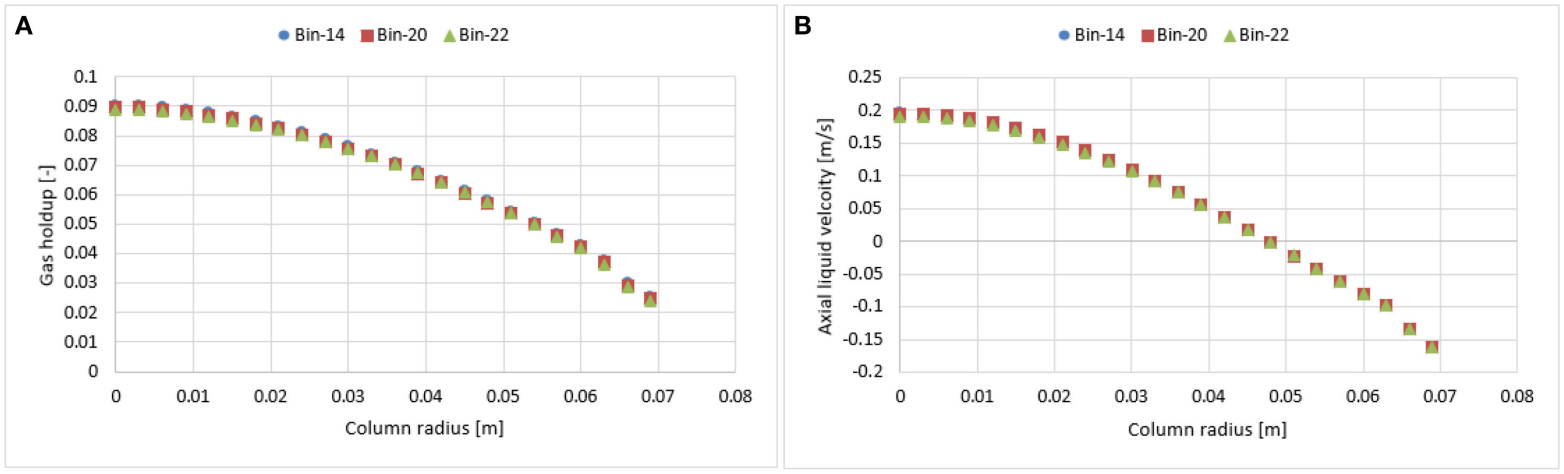
Simulated time-averaged radial profiles of the gas holdup **(A)** and axial liquid velocity **(B)** using different number of bubble classes (bins).

**Figure 2:**
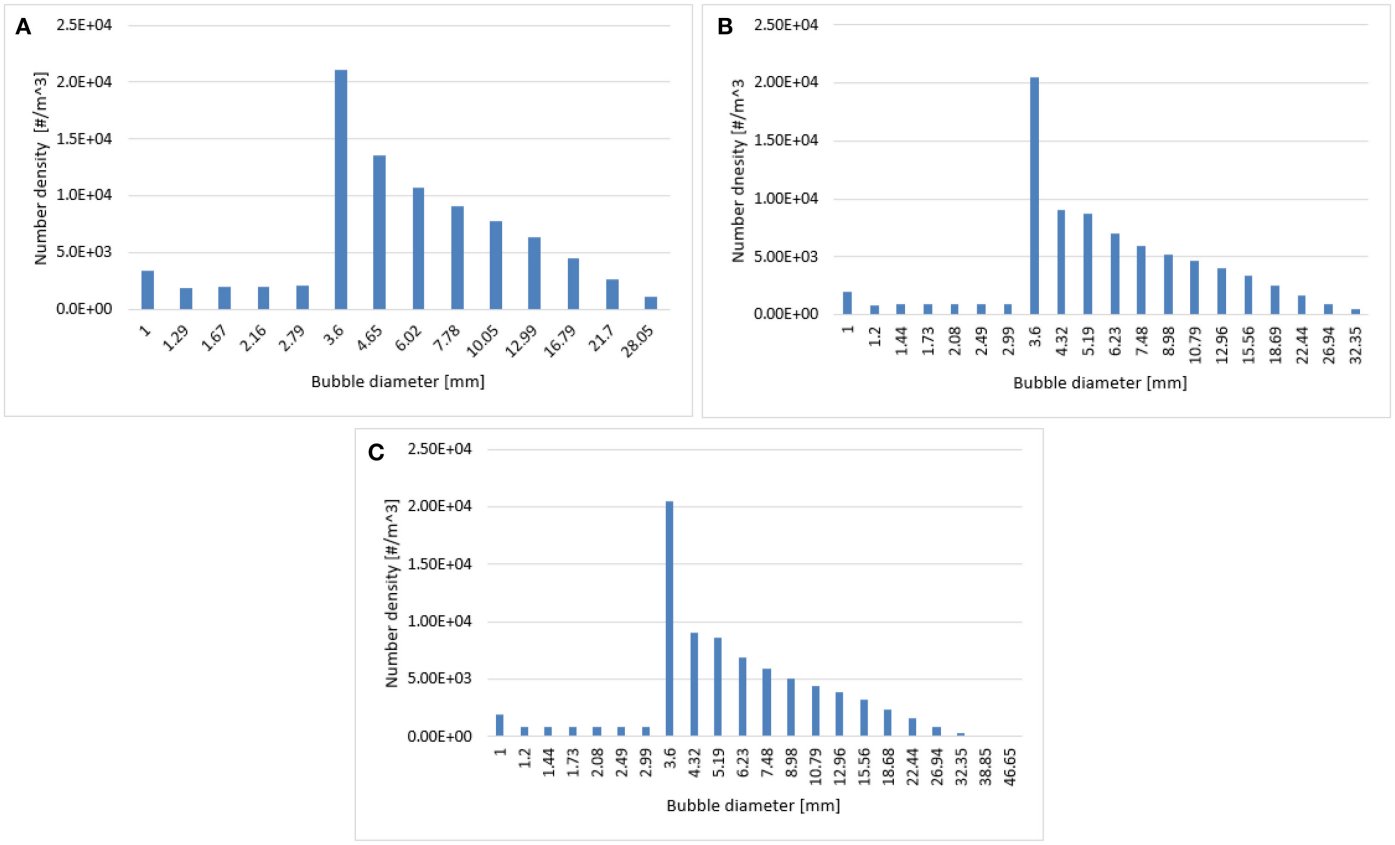
Predicted particle size distribution plotted at a 0.6 m height using **(A)** 14 bins, **(B)** 20 bins, and **(C)** 22 bins.

The effects of three different distributions of bubble classes were investigated at a 0.019 m/s superficial gas velocity. In such case, the bubble coalescence and breakup were calculated according to Luo's model. The range of bubble diameters was varied from 1 to 28 mm, 1 to 32 mm, and 1 to 46 mm hence covering all sizes of bubbles. These ranges were later divided into 14, 20, and 22 classes (bins). Figure 1 shows a comparison of the time-averaged radial profiles of the gas holdup and the axial liquid velocity obtained from simulations using a different number of bubble classes. Results show so far that there is no significant difference in the predicted radial profiles. Such behavior is reasonable because according to the predicted particle size distribution (see Figure 2) all three distributions showed a similar trend while the higher bins are almost empty in all three cases that might influence the mean-bubble size and ultimately the radial profiles. Hence, 14 bubble bins were selected for the rest of the simulations to reduce computational cost.

### Scheme analysis

**Figure 3:**
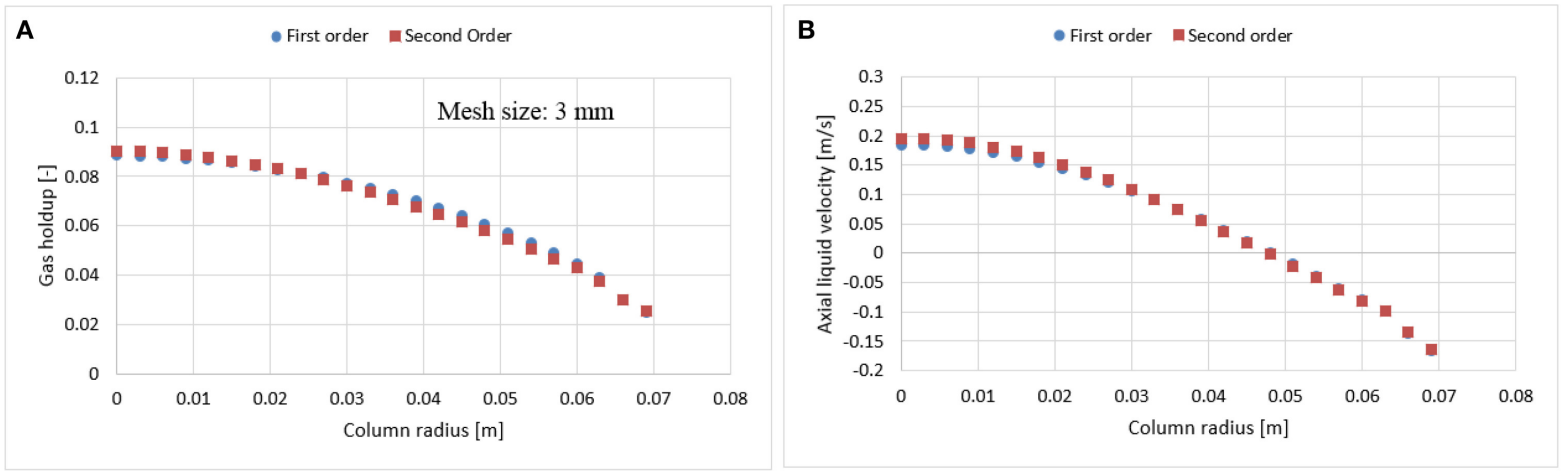
Comparison between first and second order numerical discretization scheme on gas holdup **(A)** and axial liquid velocity **(B)** using 3 mm mesh size at a 0.019 m/s superficial gas velocity.

**Figure 4:**
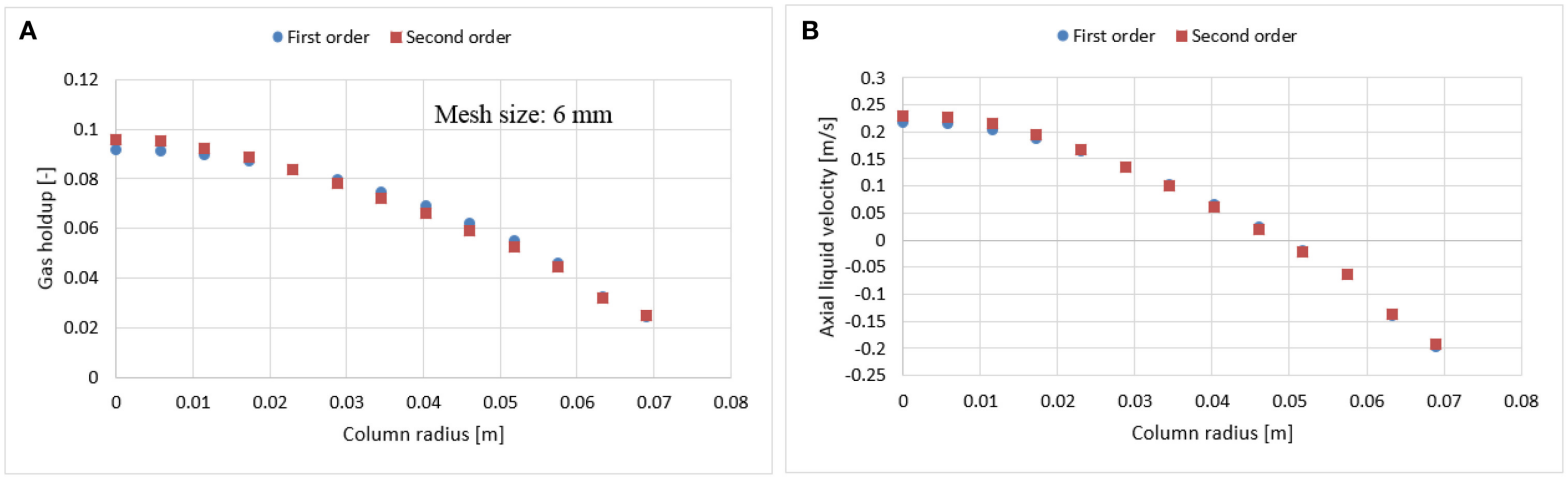
Comparison between first and second order numerical discretization scheme on gas holdup **(A)** and axial liquid velocity **(B)** using a 6 mm mesh size at 0.019 m/s superficial gas velocity.

In bubble columns, liquid re-circulation is a known phenomenon occurring for column diameters > 0.1 m (Joshi, 1980). This backflow might bring unwanted numerical diffusion in the system. To avoid such behavior, Jakobsen (2003) suggested to use a higher order scheme, which may however cause instability and convergence issues (Ansys, 2016). The latter were faced in this work for finer mesh (1.5 × 1.5 mm) with the higher order scheme. Therefore, before performing a mesh analysis, the dependency of numerical schemes (first and second order) were evaluated both on 3 and 6 mm mesh sizes (Figures 3, 4). Results show that there is no significant difference in radial profiles. However, for coarser mesh size, a slight discrepancy was observed at the core of the column where the velocity magnitude is higher as compared to near wall vicinity, which might induce the numerical diffusion and predicts slight deviation.

### Mesh and wall lubrication sensitivity

**Figure 5:**
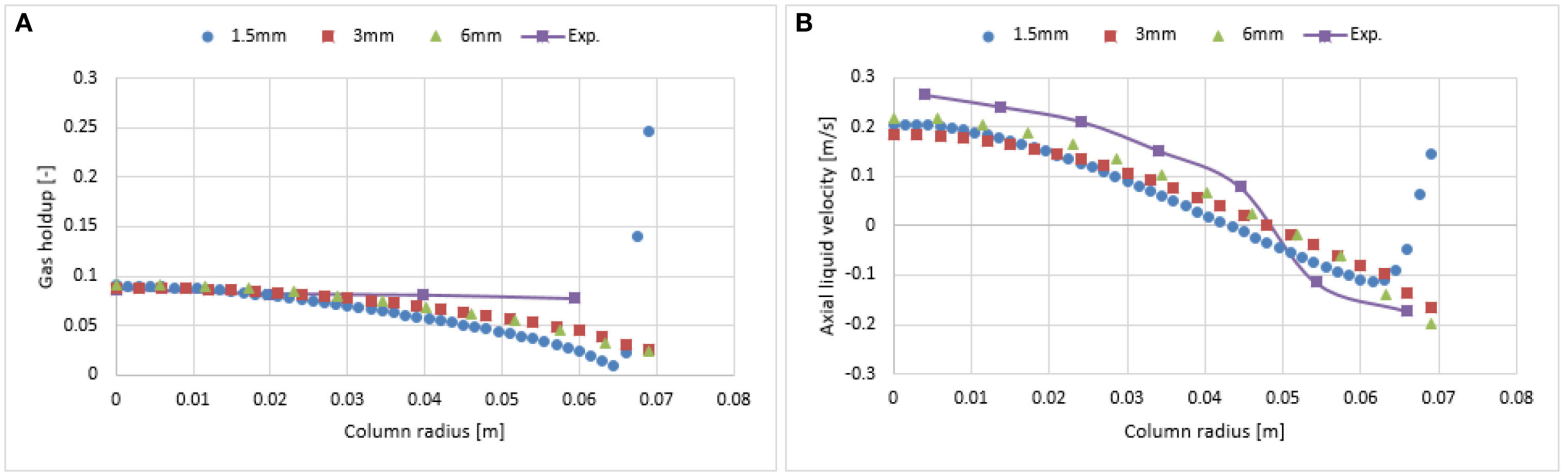
Comparison between the radial profiles of gas holdup **(A)** and axial liquid velocity **(B)** obtained from three mesh sizes and validated with experimental data from Hills (1974) at a 0.019 m/s superficial gas velocity.

**Figure 6:**
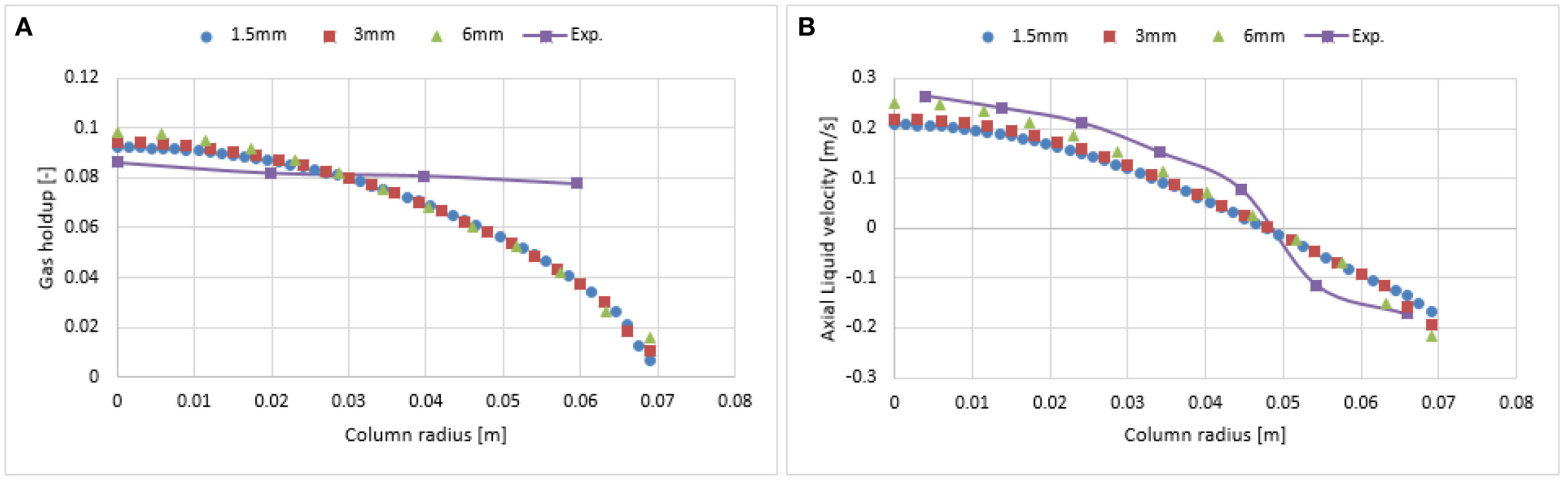
Comparison of the radial profiles of gas holdup **(A)** and axial liquid velocity **(B)** obtained from three mesh sizes using wall lubrication forces and validated with experimental data from Hills (1974) at a 0.019 m/s superficial gas velocity.

As reported in Figures 3, 4, the influence of the first and second order schemes are non-significant with regards to the radial profiles of the axial liquid velocity and the holdup (except for a slight difference at the core of the column). Therefore, a first order scheme can be used for the mesh sensitivity analysis. Hence, the investigated mesh sizes were 1.5 × 1.5 mm (fine), 3 × 3 mm (medium), and 6 × 6 mm (coarse) leading to a total number of cells for the fine, medium and coarse mesh of 41,492, 10,422, and 2,736, respectively. Figure 5 shows that the coarser mesh allows predicting a slightly higher gas hold up and axial liquid velocity due to the sharp gradient at the core of the bubble column. Simulations with finer mesh size predicted an increase of gas hold and liquid velocity near the wall. One of the possible reasons for this might be related to y^+^ values. The latter is the dimensionless wall distance, where the regime is considered as viscous (non-turbulent). This value was calculated at the cell adjacent to the wall at 0.6 m height using a continuous phase velocity. Hence, the predicted y^+^ values for the fine, medium and coarse mesh were 12.5, 26.75, and 58.29, respectively. The k-epsilon model using standard wall function depends on the y^+^ values and does not account for the turbulence parameter near the wall vicinity (viscous regime). In the case of the fine mesh, the y^+^ value is very close to the wall. Hence, the simulations predict non-realistic profiles of the gas holdup and axial liquid velocity as compared to experimental data. This discrepancy could be avoided when including the wall lubrication force (Antal et al., 1991) that pushes bubbles away from the wall (as shown in Figure 6). Results clearly show that both the 3 and 1.5 mm mesh sizes predict almost similar results following the inclusion of the wall lubrication force. Therefore, the simulations shown in the following sections were performed on a 3 mm mesh size, including the wall lubrication force. Furthermore, when including wall lubrication, the predicted axial velocity is slightly closer to experimental values. The discrepancy in gas holdup between simulations and experiment is explained in the next section. Additional investigation of wall lubrication coefficient with regards to wall distance is however beyond the scope of this study and could be the subject of future work.

### Kernels sensitivity

The PBM was solved using Luo's coalescence model as well as two different breakup kernels: Luo and Lehr. The coalescence of two bubbles in a liquid medium is often described in three basic steps. First, the bubbles collide, resulting in the trapping of a small liquid film between them. This liquid tends to drain out until the film between bubbles reaches a critical thickness. Ultimately, the thin layer of liquid ruptures and leads to the coalescence of the two bubbles. Mathematically, these bubble collisions and the contact time to layer rupture are the product of collision frequency and probability function. The bubble collision frequency includes three types of mechanism: turbulent, buoyancy, and shear-stress. In the case of the Luo coalescence kernel (Luo, 1993), the collision frequency only involves turbulent mechanism and the value related to the coalescence parameter *c*_0_ was set to 1.1107. As discussed previously, the other coalescence models presented by Lee et al. (1987) and Prince and Blanch (1990) used a similar approach but varied this coalescence parameter from 1.1 to 0.28. Xu et al. (2013, 2014) as well as Yeoh et al. (2014) reported for comparable investigations that the Luo coalescence model over-predicts the collision frequency and requires adjustment. The sensitivity analysis was performed on Luo's coalescence parameter and was tested at 1.1, 0.9, 0.5, 0.3, 0.2, and 0.1, respectively. The adjustment in the coalescence parameter was done using the user-defined-functions (UDF) and was compiled and implemented in Fluent v.17.2 accordingly.

#### Simulation with Luo's coalescence and Luo's breakup (Luo-Luo) model

**Figure 7:**
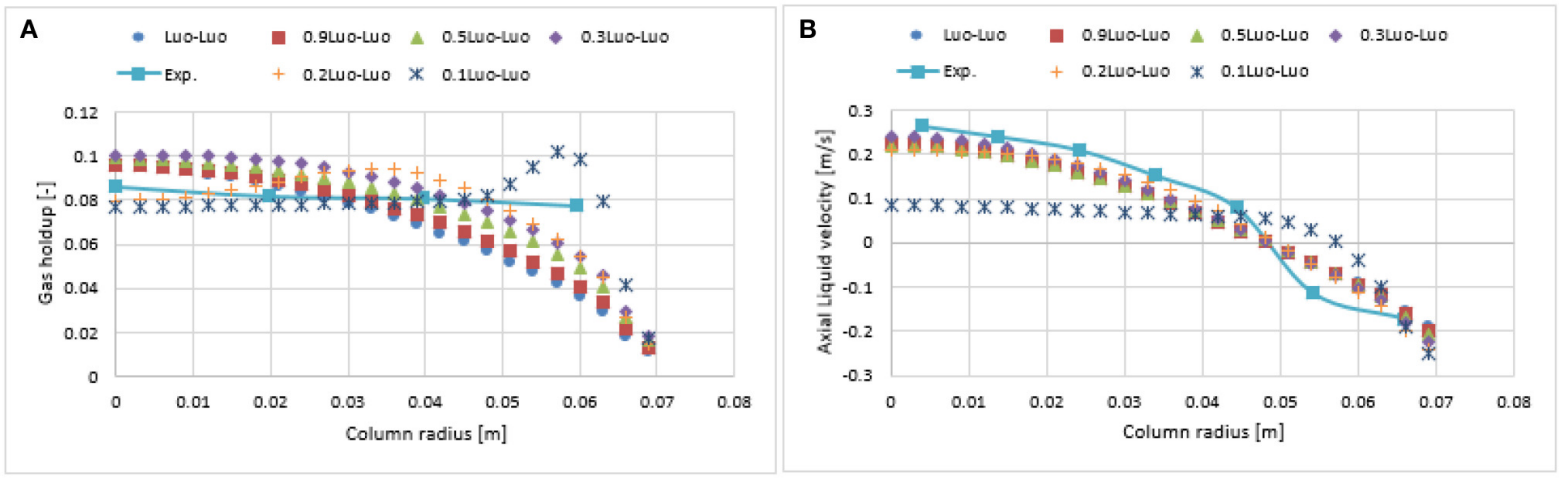
Comparison of the radial profiles of gas holdup **(A)** and axial liquid velocity **(B)** obtained from different coalescence parameter values and validated with experimental data from Hills (1974) at a 0.019 m/s superficial gas velocity.

**Figure 8:**
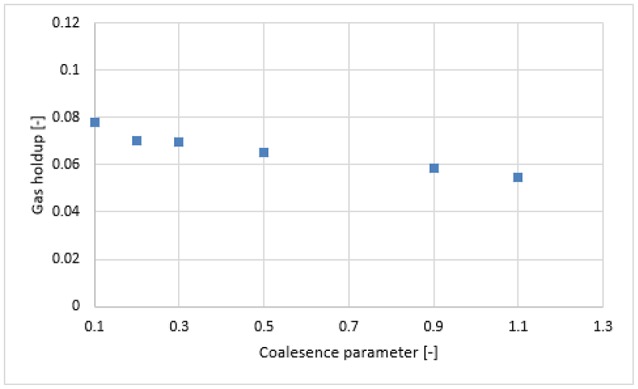
Area-weighted total gas holdup in Luo-Luo model, calculated at 0.6 m height with different coalescence parameter.

Figure 7A shows the radial profiles of the gas holdup using the Luo coalescence and Luo breakup (Luo-Luo) kernels at a 0.019 m/s superficial velocity. The Luo-Luo models predict a higher holdup at the core and a lower holdup away from the core. In addition, the shape of the simulated holdup profile is parabolic, which is similar to the data recently reported by Van Baten (2000). The latter reported that the holdup profile has a parabolic shape at a 0.019 m/s superficial velocity. This limitation of the CFD-simulation could be related to the turbulent model, which is isotropic in nature. The simulated gas holdup increased as the coalescence parameter (*c*_0_) decreased to the lowest value. One of the possible reasons is that when *c*_0_ decreases from 1.1, 0.9, 0.5, 0.3, 0.2 to 0.1, the predicted mean bubble diameter also decreases to 14.9, 13.15, 10.0, 8.4, 6.9, and 4.94 mm, respectively, leading to an increase of a gas holdup. Also, the relative difference between simulations and experiments decreases significantly with a lower value of *c*_0_ (see Table 2). The unwanted increase of the gas holdup near the vicinity of the wall especially at *c*_0_ = 0.1 is related to the lift force and is explained below. To have a clear picture of the effect of *c*_0_ on the gas holdup, the total gas holdup for the simulations was determined by taking an area-weighted integral at 0.6 m height as shown in Figure 8. As the *c*_0_ values decrease from 1.1 to 0.1, the total gas holdup increased from 5.4 to 7.8%. The calculated experimental value of the total gas holdup is 8%. Hence, at the lowest value of *c*_0_, the total gas holdup values is maximal and close to empirical value (8%). It could therefore be concluded that the modified Luo-Luo models provide total holdup results that are comparable with experiments in addition to *c*_0_ values that may require tuning from case to case.

**Table 2:** Area-weighted mean relative difference of the gas holdup profiles between experimental values of Hills (1974) and simulations using 0.019 and 0.038 m/s superficial gas velocity.

**Parameters**	**Mean-relative difference (%) at 0.019 m/s**	**Mean-relative difference (%) at 0.038 m/s**
Luo-Luo	23.14	50
0.9Luo-Luo vs. Exp.	20.57	–
0.5Luo-Luo vs. Exp.	15.93	–
0.3Luo-Luo vs. Exp.	13.3	34.91
0.2Luo-Luo vs. Exp.	13.39	29.54
0.1Luo-Luo vs. Exp.	10.85	16.6
Luo-Lehr	6.82	9.9
0.9Luo-Lehr vs. Exp.	4.11	–
0.5Luo-Lehr vs. Exp.	3.69	–
0.3Luo-Lehr vs. Exp.	3.65	–

**Figure 9:**
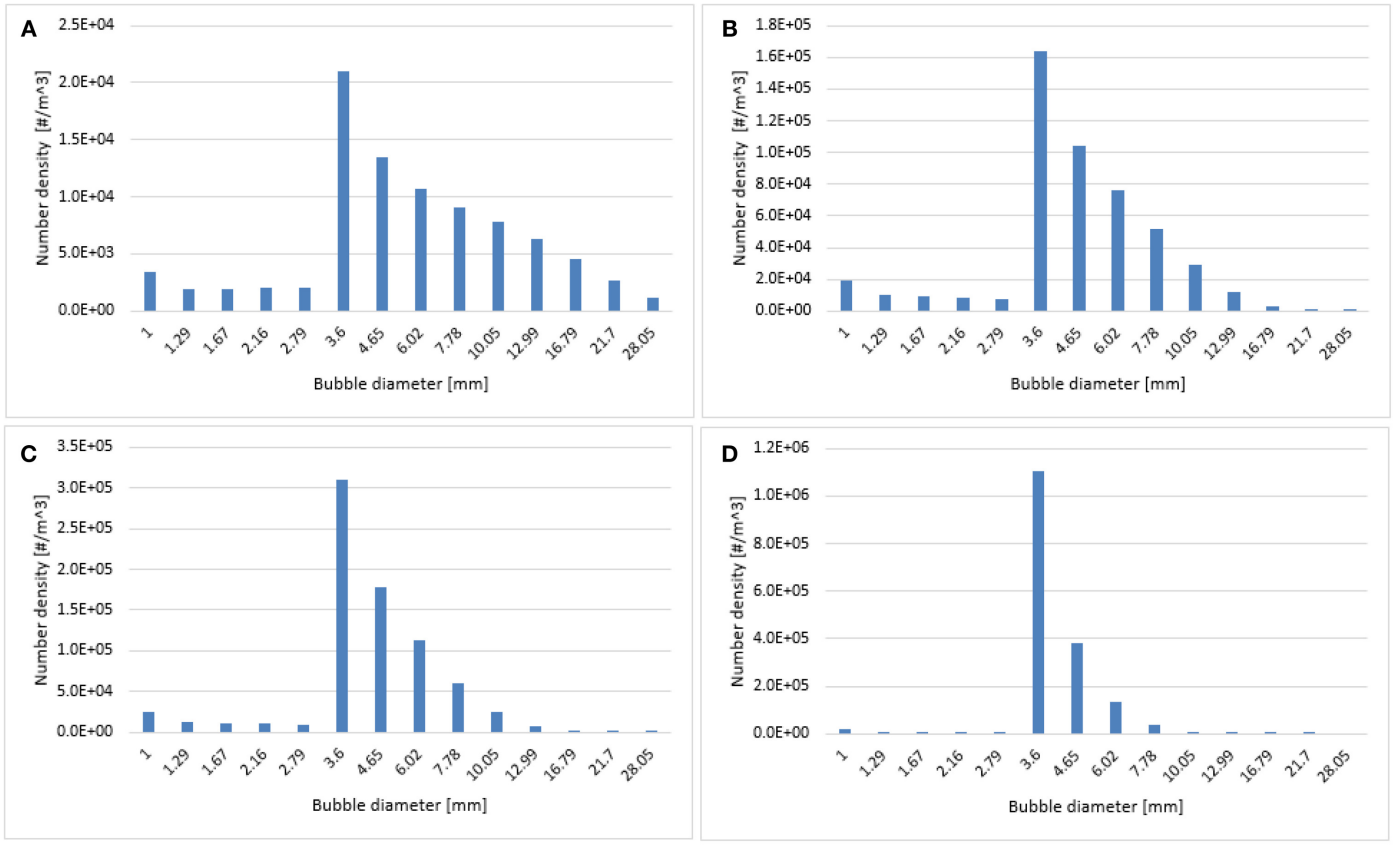
Predicted particle size distribution plotted at 0.6 m height using **(A)** Luo-Luo, **(B)** 0.3 Luo-Luo, **(C)** 0.2 Luo-Luo, and **(D)** 0.1 Luo-Luo kernels where the superficial gas velocity is 0.019 m/s.

Figure 7B shows the time-averaged axial liquid velocity for the experiments using the Luo-Luo kernels. It was observed that the effect of *c*_0_ was non-significant on the axial liquid profiles until a value of 0.2 was reached. One of the possible reasons is that the particle size distribution (see Figure 9) does not change significantly and the predicted mean bubble diameters for the Luo-Luo and 0.2 Luo-Luo kernels are 14.9 and 6.9 mm, respectively. The latter average bubble size is above the critical bubble size (*d*_*b*_ > 5.8 mm) and depicts a negative lift coefficient, therefore bubbles tend to stay at the core of the column (Tomiyama, 2004; Lucas et al., 2007). In the case of 0.1 Luo-Luo kernels, the mean bubble size (4.94 mm) is below the critical bubble size (*d*_*b*_ < 5.8 mm) and experiences positive lift coefficient, pushing the bubbles toward the wall (Tomiyama, 2004; Lucas et al., 2007). This leads to a significant decrease of the axial liquid profile as compared to the empirical values. Hence, both the gas holdup and axial liquid velocity profiles must be compared with experiments when tuning this coalescence parameter.

**Figure 10:**
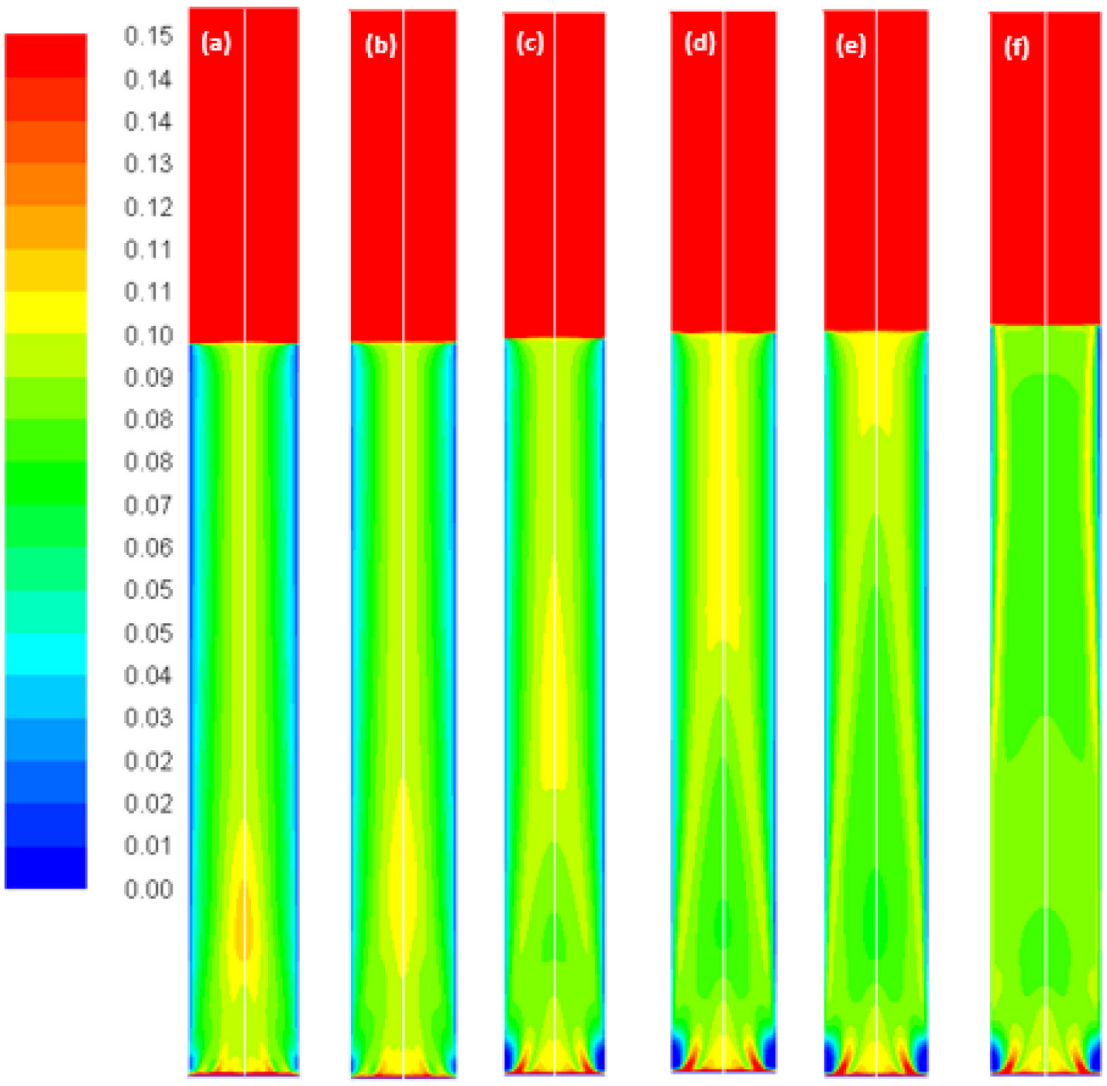
Volume-gas fractions simulated with the Luo coalescence and Luo breakup model at a Ug value of 0.019 m/s using different values of the coalescence parameter, **(a)** default value 1.1; **(b)** 0.9; **(c)** 0.5; **(d)** 0.3; **(e)** 0.2 and **(f)** 0.1.

Figure 10 depicts the time-averaged volume gas fraction in correlation with different values of *c*_0_. Results show that when moving from the gas inlet to the top of the column, the gas holdup reaches a maximum value before decreasing to a constant level. This phenomenon was clearly observed in **Figure 15**. As *c*_0_ decreases, the maximum value of gas holdup moves upward along the column (except *c*_0_ = 0.1 where the fully developed region is not reached). Therefore, it could be concluded that *c*_0_ effects the gas holdup both in the axial and radial directions.

#### Simulation with Luo's coalescence and Lehr's breakup (Luo-Lehr) model

**Figure 11:**
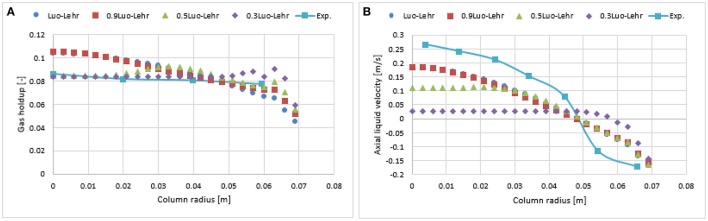
Comparison of the radial profiles of gas holdup **(A)** and axial liquid velocity **(B)** obtained from different coalescence parameter values using Luo-Lehr models and validated with experimental data from Hills (1974) at a 0.019 m/s superficial gas velocity.

**Figure 12:**
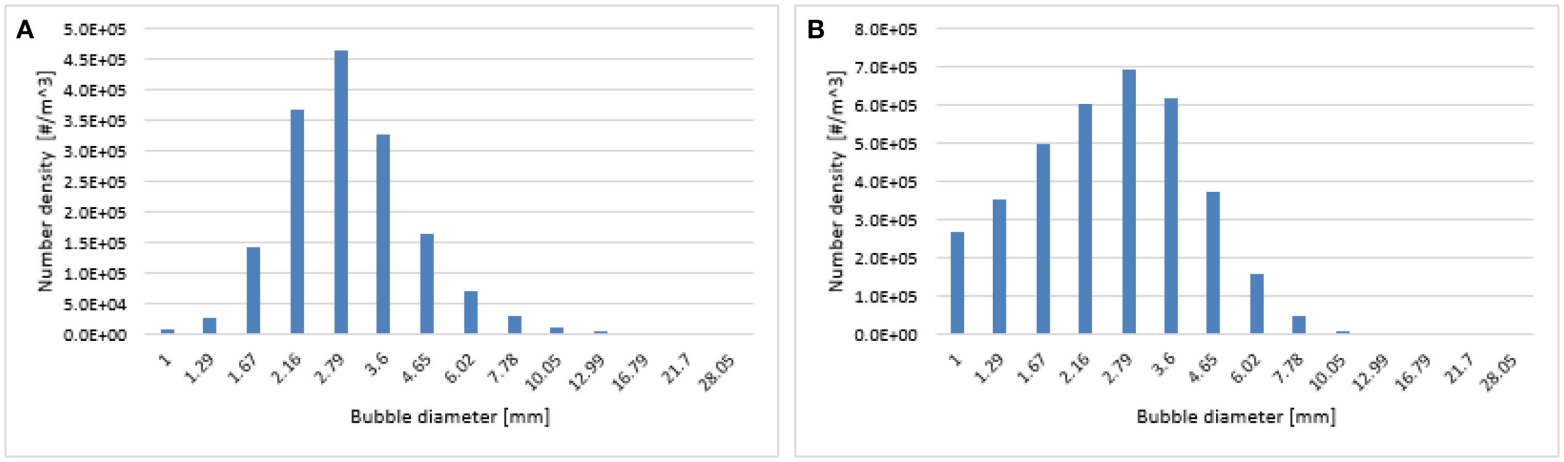
Predicted particle size distribution at a 0.6 m height using the Luo-Lehr **(A)** and 0.3 Luo-Lehr **(B)** kernels with a superficial gas velocity of 0.019 m/s.

**Figure 13:**
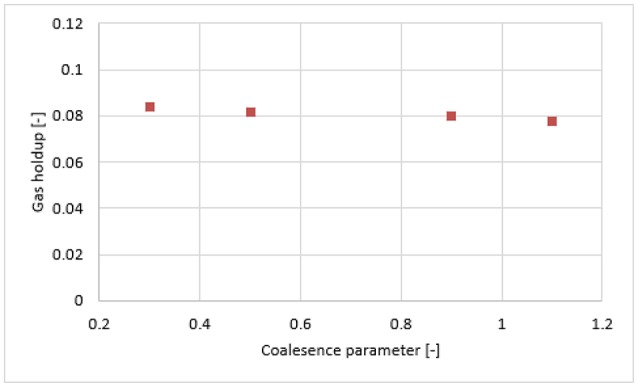
Area-weighted total gas holdup in the Luo-Lehr model, calculated at a 0.6 m height with regards to the coalescence parameter.

**Figure 14:**
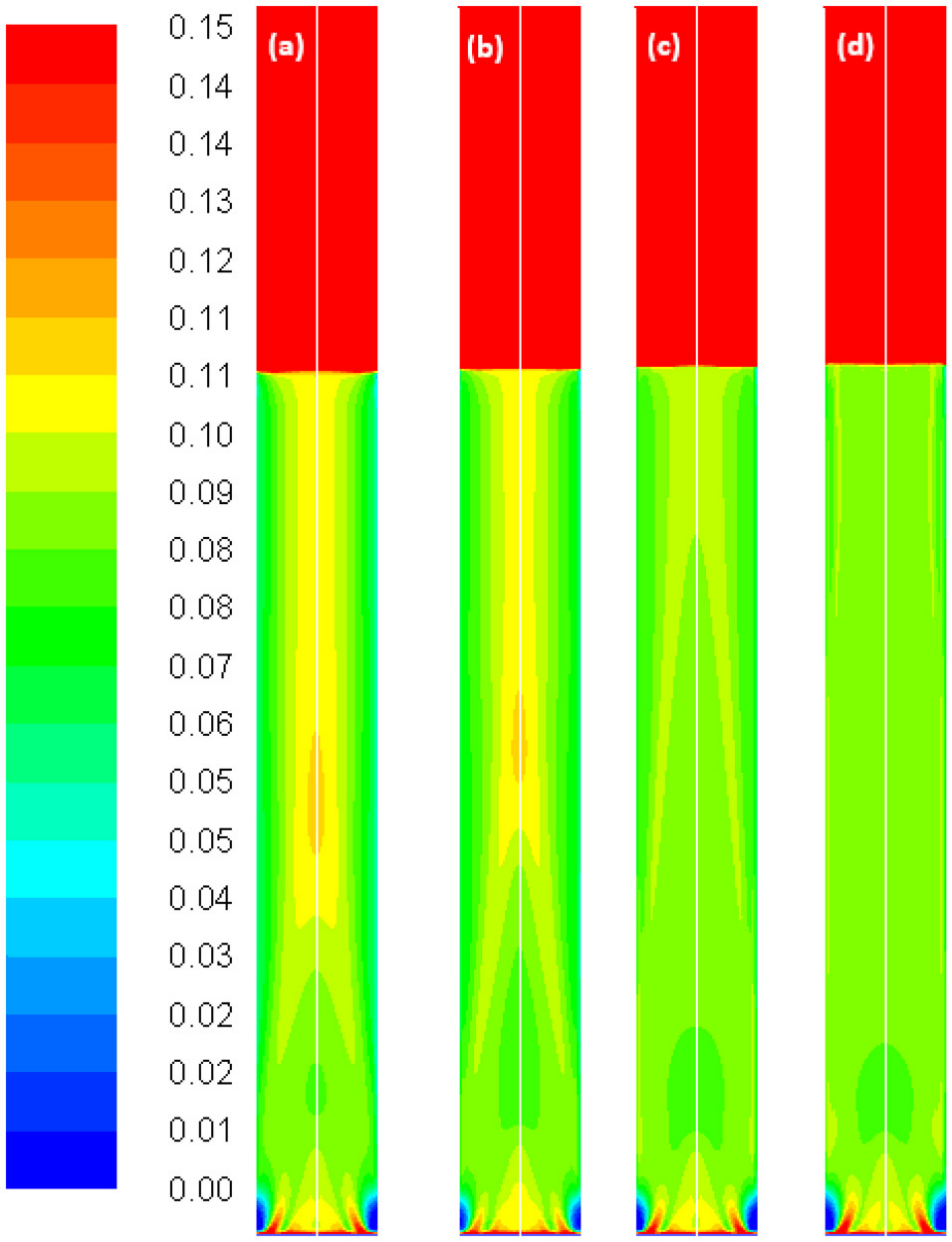
Volume-gas fractions simulated with the Luo coalescence and Lehr breakup model at a Ug value of 0.019 m/s using different values of the coalescence parameter, **(a)** default value 1.1; **(b)** 0.9; **(c)** 0.5 and **(d)** 0.3.

**Figure 15:**
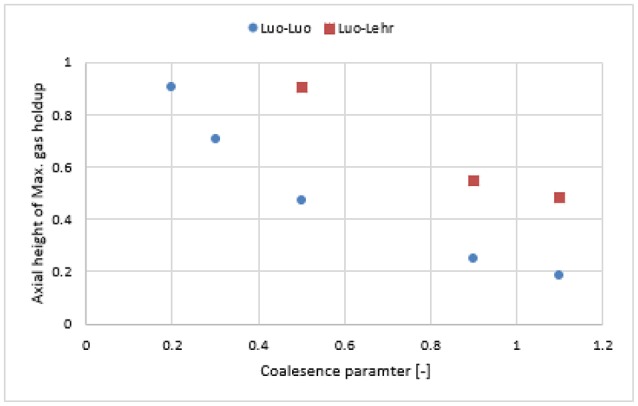
Comparison of the axial height of the maximum gas holdup obtained from Luo-Luo kernels and Luo-Lehr kernel with different values of the coalescence parameter ranging from 1.1 to 0.2.

Figure 11A shows the radial profile of the gas holdup using a combination of the Luo coalescence and Lehr breakup models (Luo-Lehr) at a 0.019 m/s superficial gas velocity. The Luo-Lehr models predict a parabolic shape of gas holdup profile similar to Luo-Luo's model, which is not consistent when compared to experimental data. As previously mentioned, such behavior might be related to limitations related to CFD calculations. Moreover, as the coalescence parameter (*c*_0_) decreases to 0.3, the predicted radial profiles become flatter and closer to experimental values, also the relative difference is lowest at 3.65% (see Table 2). However, modifications of the Luo coalescence kernel significantly under-estimate the axial liquid velocity (as shown in Figure 11B). In consequence, simulations using *c*_0_ = 0.2 and *c*_0_ = 0.1 were not performed. One of the possible reasons for such a discrepancy with the empirical values is that when *c*_0_ is shifted from its highest to lowest value (1.1 to 0.3), the particle size distribution (see Figure 12) is shifted toward the left-hand side forming smaller bubbles. The predicted mean bubble diameter in the Luo-Lehr and 0.3 Luo-Lehr kernels are 6.6 and 4.5 mm, respectively. The latter average bubble size experiences positive lift coefficient and influences the radial profile, which becomes flatter. Figure 13 shows the effect of the coalescence parameter from 1.1 to 0.3 on the Luo coalescence and Lehr breakup model in term of the total holdup. Following a decrease of the coalescence parameter from 1.1 to 0.3, the total gas holdup slightly increased from 7.7 to 8.3%. Hence, tuning the coalescence parameter doesn't lead to a significant improvement in the total holdup. Figures 14, 15 depict the time-averaged volume gas fraction with different values of coalescence parameter. The bubbles are well dispersed inside the system and the maximum value of gas holdup moves upward along the column as the *c*_0_ decreases. This behavior is consistent with the Luo-Luo kernels. However, at the lowest coalescence parameter (*c*_0_ = 0.3), the maximum gas holdup value in the Luo-Lehr kernels are not reached and the flow is in a developing stage. Because of this reason, the corresponding value for *c*_0_ (0.3) was not plotted.

#### Effect of superficial velocity

**Figure 16:**
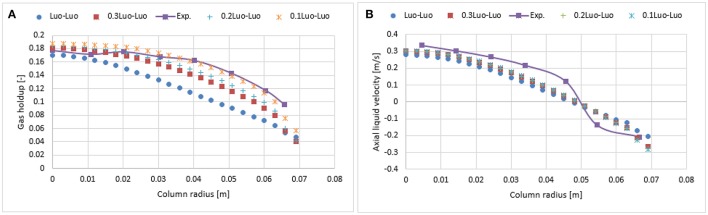
Comparison of the radial profiles of **(A)** gas holdup and **(B)** axial liquid velocity obtained from modified and non-modified Luo coalescence and Luo breakup models and validated with experimental data from Hills (1974) at a 0.038 m/s superficial gas velocity.

The impact of the higher superficial gas velocity (0.038 m/s) was studied with a combination of Luo coalescence and Luo breakup (Luo-Luo), modified Luo coalescence and Luo breakup (0.3 Luo-Luo, 0.2 Luo-Luo, and 0.1 Luo-Luo) and Luo coalescence and Lehr (Luo-Lehr) models. Figure 16A, 17A show that at an elevated superficial velocity (0.038 m/s), all combinations depict a parabolic shape holdup with regards to experimental values. The unmodified Luo-Luo models predict a significant lower holdup as compared to empirical data (50% difference, see Table 2). However, an improvement in the radial profile of gas holdup was observed as the coalescence parameter was reduced. This behavior is consistent with previously discussed results reporting that the Luo-Luo models require tuning from case to case. Furthermore, the Luo-Lehr models predict a reasonable match as compared to experiment (9.9% difference, see Table 2) without using any scaling factor, which is also consistent with previously discussed results. In Figure 16B, 17B a time-averaged radial profile of axial liquid velocity using different combinations of the models showed a similar trend and predict reasonable liquid profiles as compared to empirical values. The predicted mean-bubble size in the Luo-Luo, 0.3 Luo-Luo, 0.2 Luo-Luo, 0.1 Luo-Luo, and Luo-Lehr models was 16.04, 10.0, 8.77, 7.22, and 7.75 mm, respectively. Table 3 shows the comparison for the total holdup between CFD simulations and experimental data using a 0.019 and 0.038 m/s superficial gas velocities. Both modified Luo-Luo and Luo-Lehr models agree well with the experimental data.

**Figure 17:**
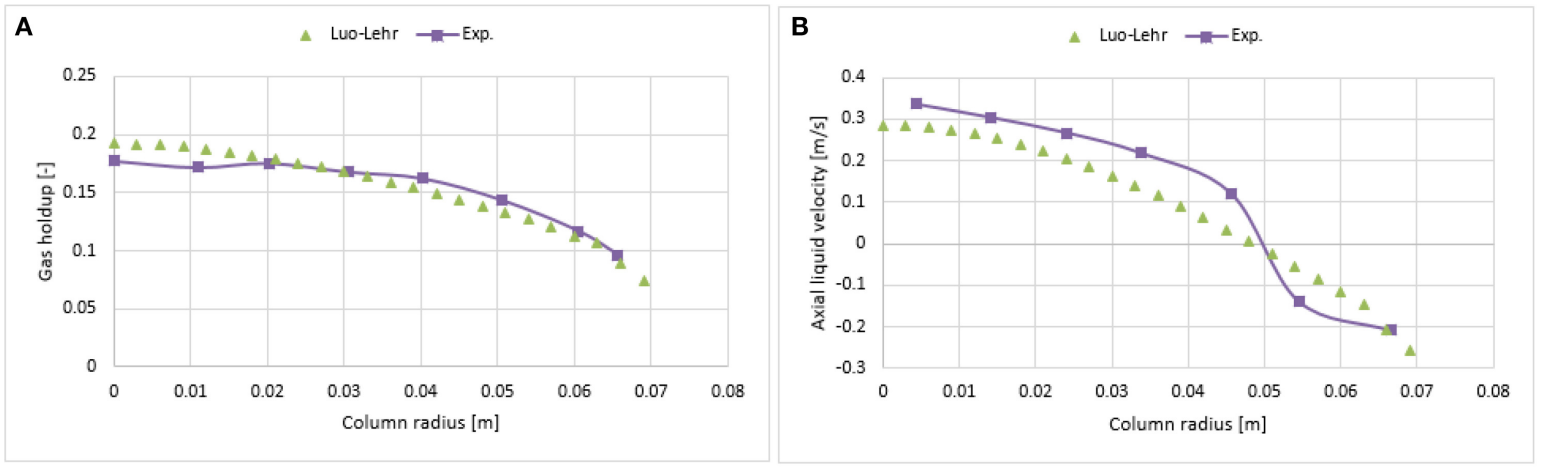
Comparison of the radial profiles of **(A)** gas holdup and **(B)** axial liquid velocity obtained from Luo coalescence and Lehr breakup models and validated with experimental data from Hills (1974) at a 0.038 m/s superficial gas velocity.

**Table 3:** Comparison of total gas holdup between CFD-simulations and experiments (Hills, 1974) at 0.019 and 0.038 m/s superficial gas velocities where the total hold up is determined by area-weighted integral of the profiles plotted at 0.6 m height.

**Results**	**Ug [m/s]**	**Total holdup [%]**
Exp	0.019	7.86
Luo-Luo	0.019	5.46
0.3Luo-Luo	0.019	6.98
0.2Luo-Luo	0.019	7.00
0.1Luo-Luo	0.019	7.79
Luo-Lehr	0.019	7.79
Exp	0.038	14.60
Luo-Luo	0.038	9.56
0.3Luo-Luo	0.038	11.85
0.2Luo-Luo	0.038	12.51
0.1Luo-Luo	0.038	13.76
Luo-Lehr	0.038	13.6

## Conclusions

In this work, 2D-axisymmetric simulations of a bubbling column were performed and the simulated time-averaged radial profiles were compared with the empirical data obtained from Hills (1974). The investigated superficial gas velocities were 0.019 and 0.038 m/s, covering the homogeneous bubbly regime. The developed model consisted of a two-fluid model coupled with a PBM. The former included the gas-liquid interface that considered the drag, lift, wall lubrication and turbulent dispersion forces. The latter involved the Luo bubble coalescence model as well as two different bubble breakup models: Luo and Lehr. From this work, the following conclusions could hence be formulated:

The sensitivity analysis of the bubble classes was performed using 14, 20, and 22 bins and it was shown that the solution was independent of the bubble classes. Hence, the lowest number of bubble classes were selected to reduce computational cost.

Verification of the numerical schemes was performed using first and second orders and results showed that numerical schemes had no significant influence on the predicted radial profiles of gas holdup and axial liquid velocity. However, at the center of the column, a slight discrepancy was observed, which might be related to numerical diffusion.

Mesh sensitivity was conducted on 1.5 mm (finer), 3 mm (medium), and 6 mm (coarse) mesh sizes. The predicted axial liquid profile of coarse (6 mm) mesh size differed from medium and fine mesh size, and hence was ignored. The fine meshing showed a non-realistic behavior near the wall without the inclusion of wall lubrication force, which might be related to the y^+^ value of 12.5 and once introduced, there was no significant difference between fine- and medium-sized mesh. In addition, the predicted axial liquid velocity slightly improved at the core of the bubble column.

The combination of the Luo coalescence and Luo breakup kernels (Luo-Luo) was shown to under-predict the gas holdup both at a 0.019 and 0.038 m/s superficial gas velocity. The gas holdup was increased to a maximum when the coalescence parameter was reduced. However, at the lowest Ug and the *c*_0_ (=0.1) values, the predicted velocity profile was far away from the experimental values. It is thus recommended to tune the coalescence parameter when using the Luo-Luo kernels and both the holdup and axial liquid profiles should be considered for validation purposes.

Simulations using a combination of the Luo coalescence and Lehr breakup kernels (Luo-Lehr) predicted a closer holdup both for the 0.019 and 0.038 m/s superficial velocities when compared with experiments. Scaling of coalescence parameter, in combination with the Lehr model leads to no significant improvement in the gas holdup. Furthermore, a decrease of the coalescence parameter significantly influences the axial liquid profile that under-predicts the profile compared to experiments. Results have shown that it is better to use Luo-Lehr kernels without any modification of the coalescence parameter.

## Author contributions

AS: Wrote and drafted the article. MB, TM: Helped in developing the model and provided the technical guidelines. JL: Reviewed and approved the article, managed the research, reviewed the results and provided the technical guidelines.

### Conflict of interest statement

The authors declare that the research was conducted in the absence of any commercial or financial relationships that could be construed as a potential conflict of interest.

## Figures and Tables

**Table:** 

*c*_0_	Coalescence parameter
*C*_1_	Constant equals 1.44
*C*_2_	Constant equals 1.92
*C*_*D*_	Drag coefficient
*C*_*D,vis*_	Drag coefficient for the viscous regime
*C*_*D,dis*_	Drag coefficient for the distorted regime
*C*_*D,cap*_	Drag coefficient for the capped regime
*c*_*f*_	Coefficient of surface increment due to bubble breakage
*C*_*L*_	Lift coefficient
*C*_*w*1_, *C*_*w*2_	Constant values -0.01 and 0.05
*C*_*WL*_	Wall lubrication coefficient
*C*_*TD*_	User modifiable constant equals 1
*D*_*t,pq*_	Scalar fluid particulate dispersion tensor
*d*_*b*_	Bubble size, m
*d*_*h*_	Maximum horizontal length of the deformed bubble, m
*d*_*p*_	Dispersed phase mean diameter, m
*d*_*i*_, *d*_*j*_	Diameter of bubble of size i and j, m
*d*_max_, *d*_min_	Maximum/Minimum diameter of bubble, m
*Eo*′, *Eo*	Modified Eotvos number and Eotvos number
***F***_*pq*_	Inter-phase momentum exchange term between disperse phase p and continuous phase q, kg/m^2^·s^2^
***F***_*D*_	Drag force, kg/m^2^·s^2^
***F***_*L*_	Lift force, kg/m^2^·s^2^
***F***_*TD*_	Turbulent dispersion force, kg/m^2^·s^2^
***F***_*WL*_	Wall lubrication force, kg/m^2^·s^2^
*f*	Volume fraction of daughter bubble
*G*	Production of kinetic energy
***g***	Gravitational force, m/s^2^
*K*_*pq*_	Inter-phase momentum coefficient
*k*	Mean turbulent kinetic energy per unit mass, m^2^/s^2^
*n*_*i*_	Number density of bubble size i, #/m^3^
***n***_*w*_	Unit normal pointing away from the wall
*P*	Pressure, Pa
*Re*	Reynolds number
*r*	Ratio factor for bubble volume
*t*	Time, s
*t*_*c*_	Bubble contact time, s
*t*_*I*_	Film drainage time, s
***u***	Phase velocity, m/s
Ug	Superficial gas velocity, m/s
*v*_*i*_	Volume of ith bubble class, m^3^
*We*_*crit*_	Critical Weber number
*y*_*w*_	Distance to the nearest wall, m
*y*^+^	Dimensionless wall distance
**Greek letters**	
α	Phase fraction
ε	Energy dissipation rate, m^2^/s^3^
ξ	Ratio of eddy size to parent bubble
ρ	Density, kg/m^3^
ρ_*pq*_	Absolute value of the density difference between disperse phase p and continuous phase q
σ	Surface tension, N/m
σ_*k*_	Constant equals 1
σ_ε_	Constant equals 1.3
Ω_*B*_	Breakup rate, 1/m^3^s
Ω_*C*_	coalescence rate, 1/m^3^s
μ	Shear viscosity of phase, kg/m
μ_*t*_	turbulent viscosity, kg/m
**Subscripts**	
*q*	Continuous phase
*p*	Disperse phase
*m*	Mixture phase
